# Efficacy and safety of first-line avelumab in patients with advanced non-small cell lung cancer: results from a phase Ib cohort of the JAVELIN Solid Tumor study

**DOI:** 10.1136/jitc-2020-001064

**Published:** 2020-09-08

**Authors:** Claire F Verschraegen, Guy Jerusalem, Edward F McClay, Nicholas Iannotti, Charles H Redfern, Jaafar Bennouna, Franklin L Chen, Karen Kelly, Janice Mehnert, John C Morris, Matthew Taylor, David Spigel, Ding Wang, Hans Juergen Grote, Dongli Zhou, Neru Munshi, Marcis Bajars, James L Gulley

**Affiliations:** 1 Division of Medical Oncology, The Ohio State University James Cancer Hospital, Columbus, Ohio, USA; 2 Department of Medical Oncology, CHU Sart Tilman Liege and Liege University, Liege, Belgium; 3 Institute for Melanoma Research & Education, California Cancer Associates for Research & Excellence, Encinitas, California, USA; 4 Hematology Oncology Associates of the Treasure Coast, Port St. Lucie, Florida, USA; 5 Sharp Healthcare, San Diego, California, USA; 6 Department of Pneumology, Thoracic Oncology, University Hospital Centre Nantes, Nantes, France; 7 Novant Health Oncology Specialists, Winston-Salem, North Carolina, USA; 8 Internal Medicine, University of California Davis Comprehensive Cancer Center, Sacramento, California, USA; 9 Rutgers Cancer Institute of New Jersey, New Brunswick, New Jersey, USA; 10 University of Cincinnati Cancer Institute, Cincinnati, Ohio, USA; 11 Providence Cancer Center, Portland, Oregon, USA; 12 Sarah Cannon Research Institute, Nashville, Tennessee, USA; 13 Henry Ford Medical Center, Detroit, Michigan, USA; 14 Merck KGaA, Darmstadt, Germany; 15 Merck Serono Pharmaceutical R&D Co, Beijing, China; an affiliate of Merck KGaA, Darmstadt, Germany; 16 EMD Serono Research & Development Institute, Inc, Billerica, Massachusetts, USA; a business of Merck KGaA, Darmstadt, Germany; 17 Genitourinary Malignancies Branch and Laboratory of Tumor Immunology and Biology, Center for Cancer Research, National Cancer Institute, National Institutes of Health, Bethesda, Maryland, USA

**Keywords:** programmed cell death 1 receptor, immunotherapy, clinical trials as topic

## Abstract

**Introduction:**

Avelumab, an antiprogrammed death ligand-1 antibody, is approved as a monotherapy for treatment of metastatic Merkel cell carcinoma and advanced urothelial carcinoma, and in combination with axitinib for advanced renal cell carcinoma. We report the efficacy and safety of first-line avelumab in advanced non-small cell lung cancer (NSCLC).

**Methods:**

In a phase I expansion cohort of the JAVELIN Solid Tumor trial, patients with treatment-naive, metastatic, or recurrent NSCLC received 10 mg/kg avelumab intravenously every 2 weeks. Endpoints included best overall response, duration of response (DOR), progression-free survival (PFS), overall survival (OS), and safety.

**Results:**

Overall, 156 patients were enrolled and treated. Median duration of follow-up was 18.6 months (range, 15 to 23 months). The objective response rate was 19.9% (95% CI, 13.9 to 27.0), including complete response in 3 (1.9%) and partial response in 28 (17.9%). Median DOR was 12.0 months (95% CI, 6.9 to not estimable). Median PFS was 4.0 months (95% CI, 2.7 to 5.4) and the 6-month PFS rate was 38.5% (95% CI, 30.7 to 46.3). Median OS was 14.1 months (95% CI, 11.3 to 16.9) and the 12-month OS rate was 56.6% (95% CI, 48.2 to 64.1). Treatment-related adverse events (TRAEs) occurred in 107 patients (68.6%), including grade ≥3 TRAEs in 19 (12.2%). Immune-related adverse events and infusion-related reactions occurred in 31 (19.9%) and 40 patients (25.6%), respectively. No treatment-related deaths occurred.

**Conclusion:**

Avelumab showed antitumor activity with a tolerable safety profile as a first-line treatment in patients with advanced NSCLC. These data support further investigation of avelumab in the phase III JAVELIN Lung 100 study.

**Trial registration details:**

ClinicalTrials.gov NCT01772004; registered January 21, 2013.

## Introduction

Monoclonal antibodies targeting immune checkpoint proteins are established treatments for metastatic non-small cell lung cancer (NSCLC). In 2016, the antiprogrammed cell death protein-1 (PD-1) antibody pembrolizumab was approved as first-line monotherapy for patients with metastatic NSCLC without targetable epidermal growth factor receptor (*EGFR*) or anaplastic lymphoma kinase (*ALK*) gene defects and with ≥50% programmed death ligand 1 (PD-L1) expression on tumor cells.^1 2^ Based on results of the phase III KEYNOTE-042 study, approval of pembrolizumab monotherapy in the first-line setting was expanded in the USA for patients with ≥1% PD-L1 expression on tumor cells.^3^ By contrast, in a phase III trial of first-line nivolumab (anti-PD-1) monotherapy versus platinum-based chemotherapy in patients with advanced NSCLC with ≥5% PD-L1 expression on tumor cells, nivolumab did not significantly improve progression-free survival (PFS) or overall survival (OS).^4^ More recently, immune checkpoint inhibitors (pembrolizumab and atezolizumab) have been assessed in combination with chemotherapy in the first-line NSCLC setting and have shown superior efficacy compared with chemotherapy alone in several randomized trials.^5–7^

Avelumab, a human anti-PD-L1 immunoglobulin G1 antibody with a wild-type Fc region, has been shown in preclinical models to induce antitumor activity via adaptive and innate effector cells.^8 9^ Avelumab has been approved in some countries as monotherapy for metastatic Merkel cell carcinoma, as monotherapy for advanced urothelial carcinoma that has not progressed with platinum-containing chemotherapy (first-line maintenance therapy) or following disease progression, and in combination with axitinib for first-line treatment of advanced renal cell carcinoma.^10 11^ In previous clinical trials, avelumab has demonstrated clinical activity and a tolerable safety profile in patients with various other tumor types, including platinum-treated NSCLC.^12–16^ Here, we report efficacy and safety data from a phase I expansion cohort of the JAVELIN Solid Tumor trial in which patients with advanced NSCLC received first-line avelumab. Preliminary findings from this cohort led to the initiation of the phase III JAVELIN Lung 100 study (NCT02576574) of first-line avelumab versus platinum-based doublet chemotherapy in patients with PD-L1-positive NSCLC.^17^

## Materials and methods

### Study design and treatment

JAVELIN Solid Tumor (NCT01772004) is an international, multicohort, open-label phase I trial. In this phase Ib NSCLC expansion cohort, patients, who were unselected for PD-L1 expression, had histologically confirmed stage IV (per International Association for the Study of Lung Cancer classification, seventh edition)^18^ or recurrent NSCLC, no prior treatment for metastatic or recurrent disease, and no activating *EGFR* mutation or *ALK* translocation/rearrangement (tumors with non-squamous cell histology were tested if mutational status was unknown). General eligibility criteria for the JAVELIN Solid Tumor trial have been reported previously.^12^

### Procedures and assessments

Patients received avelumab 10 mg/kg by intravenous infusion every 2 weeks until disease progression, unacceptable toxicity, or other criteria for withdrawal were met. Patients were permitted to continue treatment despite progression according to the investigator’s decision and in agreement with the patient if no new symptoms appeared, existing symptoms did not worsen, Eastern Cooperative Oncology Group (ECOG) performance status did not decrease, and the investigator did not consider it necessary to administer a salvage therapy. Dose reductions were not permitted. Premedication with an antihistamine (diphenhydramine or equivalent) and acetaminophen was given 30 to 60 min before each infusion. Treatment was permanently discontinued for any grade ≥3 adverse event (AE) except for specified transient AEs (reported previously).^12 13^ Grade 2 AEs were managed by treatment delays of ≤2 subsequent omitted doses; events that did not resolve to grade ≤1 or recurred resulted in permanent discontinuation of avelumab. Clinical activity and safety were analyzed in all patients who received ≥1 dose of avelumab.

Tumor assessments were performed every 6 weeks for the first year and every 12 weeks thereafter by investigators according to Response Evaluation Criteria in Solid Tumors 1.1 (RECIST 1.1) and modified immune-related response criteria.^19^ Safety was assessed every 2 weeks at each visit, and AEs were graded according to National Cancer Institute Common Terminology Criteria for Adverse Events V.4.0. Immune-related AEs (irAEs) were identified using a prespecified list of Medical Dictionary for Regulatory Activities (MedDRA)-preferred terms followed by comprehensive medical review. Infusion-related reactions (IRRs) were identified using an expanded definition that included both a prespecified list of MedDRA-preferred terms (IRR, drug hypersensitivity, or hypersensitivity reaction) that occurred post infusion within 48 hours, and additional signs or symptoms that occurred on the day of infusion and resolved within 2 days.

PD-L1 expression was assessed using a proprietary immunohistochemistry assay (PD-L1 IHC 73-10 pharmDx; Dako, Carpinteria, California). In previous studies comparing the 73-10 PD-L1 assay with the 22C3 assay used in pembrolizumab trials, the 73-10 assay showed greater sensitivity, and the ≥80% PD-L1 cut-off for the 73-10 assay was found to be comparable to the ≥50% PD-L1 cut-off for the 22C3 assay (manuscript in press).^20 21^ PD-L1-positive status was predefined as PD-L1 expression of any intensity on ≥1% of tumor cells; PD-L1 expression status was also assessed using cut-offs of ≥50% and ≥80% in post hoc analyzes.

Prespecified endpoints assessed in this expansion cohort included confirmed best overall response, duration of response (DOR), and PFS based on investigator assessment according to RECIST 1.1, best overall response based on investigator assessment according to modified immune-related response criteria, OS, PD-L1 expression, and safety (all secondary endpoints in the overall JAVELIN Solid Tumor trial protocol).

### Statistical analysis

Planned enrollment was 150 patients, which was based on the anticipated sample size required to estimate and provide 95% Clopper-Pearson CIs for potential objective response rates (ORRs). Safety data were summarized using descriptive statistics. Time-to-event endpoints were estimated using the Kaplan-Meier method, while 95% CIs for medians were calculated using the Brookmeyer-Crowley method. P values for the association between PD-L1 status and ORRs were determined using the Fisher’s exact test. Comparisons between other subgroups were not prespecified and are reported descriptively.

## Results

### Patient characteristics and disposition

Between March 18, 2015, and November 19, 2015, 156 patients were enrolled from seven countries in North America, Europe, and Asia (table 1). The median age was 69.5 years (range, 41 to 90 years), 83 patients (53.2%) were male, and most patients (108 (69.2%)) had an ECOG performance status of 1. Tumor histology was squamous in 46 patients (29.5%) and non-squamous in 110 (70.5%). A total of 139 patients (89.1%) were ever smokers (current or previous) and 17 (10.9%) had never smoked. PD-L1 expression was evaluable in 111 patients (71.2%), of whom 88 (79.3%) had PD-L1-positive tumors based on a ≥1% cut-off. *EGFR* and *ALK* mutation status were unknown in 18 (11.5%) and 16 (10.3%), respectively. At data cut-off (February 15, 2017), the median follow-up was 18.6 months (range, 15 to 23 months). Patients received a median of 12 avelumab infusions (range, 1 to 49) over a median duration of 5.5 months (range, 0.5 to 22.5 months). At data cut-off, 26 patients (16.7%) remained on study treatment. Reasons for permanent treatment discontinuation were disease progression (82 (52.6%)), AEs (24 (15.4%)), withdrawal of consent (9 (5.8%)), death (9 (5.8%)), and other reasons (6 (3.8%)).

**Table 1:** Patient demographics and baseline characteristics

Characteristic	N=156
Age	
Median (range), years	69.5 (41 to 90)
<65, n (%)	51 (32.7)
≥65, n (%)	105 (67.3)
Sex, n (%)	
Male	83 (53.2)
Female	73 (46.8)
Geographic region, n (%)	
North America	127 (81.4)
Europe	25 (16.0)
Asia	4 (2.6)
Race, n (%)	
White	124 (79.5)
Black or African American	12 (7.7)
Asian	6 (3.8)
Other	14 (9.0)
ECOG PS, n (%)	
0	46 (29.5)
1	108 (69.2)
2*	2 (1.3)
Smoking status, n (%)	
Never used	17 (10.9)
Regular user	29 (18.6)
Occasional user	2 (1.3)
Former user	108 (69.2)
Time since first diagnosis, median (range), months	2.0 (0.02 to 143.5)
Time since diagnosis of metastatic disease, median (range), months†	1.5 (0.2 to 92.0)
Tumor histology, n (%)	
Squamous cell carcinoma	46 (29.5)
Non-squamous cell carcinoma	110 (70.5)
*EGFR* mutation status, n (%)	
Wild type	137 (87.8)
Mutant‡	1 (0.6)
Unknown	18 (11.5)
*ALK* mutation status, n (%)	
Wild type	140 (89.7)
Mutant	0
Unknown	16 (10.3)
*KRAS* mutation status, n (%)	
Wild type	6 (3.8)
Mutant	10 (6.4)
Unknown	140 (89.7)
PD-L1 expression ≥1% of tumor cells, n (%)	
Positive	88 (56.4)
Negative	23 (14.7)
Not evaluable§	45 (28.8)
PD-L1 expression ≥50% of tumor cells, n (%)	
Positive	53 (34.0)
Negative	58 (37.2)
Not evaluable§	45 (28.8)
PD-L1 expression ≥80% of tumor cells, n (%)	
Positive	38 (24.4)
Negative	73 (46.8)
Not evaluable§	45 (28.8)

*Both patients had an ECOG PS of 1 at baseline, which had increased to 2 at the first dose of study treatment.

†Data missing for six patients.

‡This patient was permitted to enroll following discussions between the investigator and the sponsor based on expected resistance to available tyrosine kinase inhibitor therapy.

§Reasons for PD-L1 expression not being evaluable included tumor sample containing insufficient tumor cells (<100), non-evaluable sample type (eg, cytology specimen), and no tumor tissue available for analysis.

ALK, anaplastic lymphoma kinase; ECOG PS, Eastern Cooperative Oncology Group performance status; EGFR, epidermal growth factor receptor; PD-L1, programmed death ligand-1.

### Efficacy

**Figure 1:**
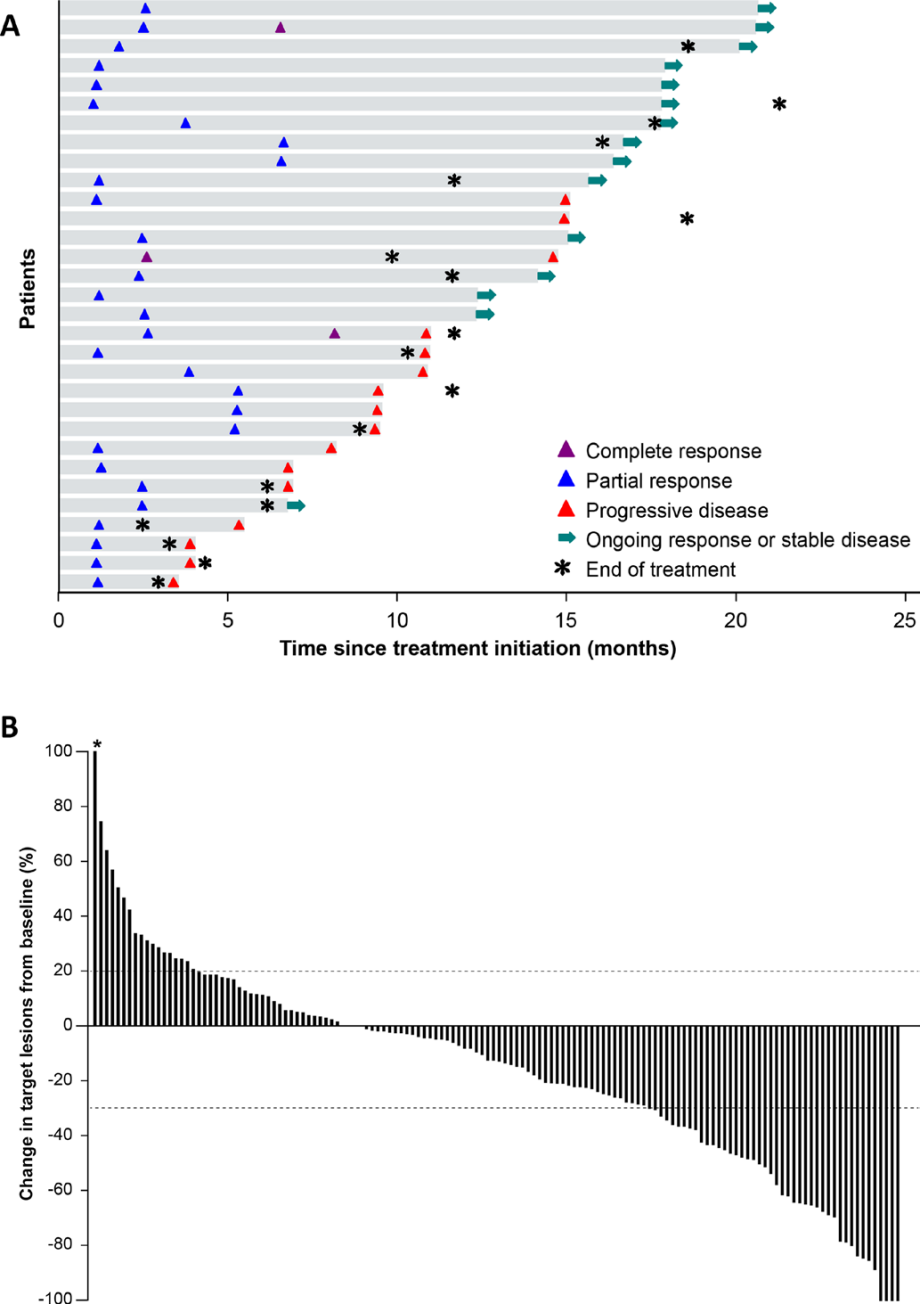
(A) Time to and duration of response in patients with confirmed complete response or confirmed partial response (n=31). The first per-protocol scan was performed after 6 weeks for the first tumor assessment (week 7). Median follow-up was 18.6 months (range, 15 to 23 months). (B) Best change from baseline in target lesions in evaluable patients (patients with a baseline and at least one post-baseline lesion assessment; n=142). *Patient with 415% increase in tumor diameter imputed with a cap of 100%.

Of 156 patients, 3 (1.9%) had a confirmed complete response (CR) and 28 (17.9%) had a confirmed partial response (PR), resulting in an ORR of 19.9% (95% CI, 13.9% to 27.0%); 17 patients (10.9%) were not evaluable for response per RECIST (missing evaluations or not assessable; table 2). ORRs were observed in 17.4% (95% CI, 7.8% to 31.4%) of patients with squamous and 20.9% (95% CI, 13.7% to 29.7%) of patients with non-squamous histology. ORRs in ever smokers and never smokers were 20.9% (95% CI, 14.4% to 28.6%) and 11.8% (95% CI, 1.5% to 36.4%), respectively. In patients who had unknown *EGFR* or *ALK* mutation status, ORRs were 16.7% (95% CI, 3.6% to 41.4%) and 18.8% (95% CI, 4.0% to 45.6%), respectively. Of the three patients who had a CR, two had a preceding PR. Response was ongoing in 15 of 31 patients at data cut-off (figure 1A). The median time to response was 11.4 weeks (range, 5.1 to 29.6 weeks) and the median DOR in patients with confirmed responses was 12.0 months (95% CI, 6.93 months to not estimable). Of 142 patients who were evaluable for changes in target lesions (ie, those with a baseline and on-study tumor assessment available), 93 (65.5%) had a reduction in tumor size of any level, while 43 (30.3%; including 12 patients with unconfirmed responses) had ≥30% reduction (figure 1B and online additional file 1), with no notable trends based on tumor histology or smoking status (online additional file 2). The median PFS in all patients was 4.0 months (95% CI, 2.7 to 5.4 months) and the 6-month PFS rate was 38.5% (95% CI, 30.7% to 46.3%) (online additional file 3). The median OS was 14.1 months (95% CI, 11.3 to 16.9 months) and the 12-month OS rate was 56.6% (95% CI, 48.2% to 64.1%) (online additional file 3).

10.1136/jitc-2020-001064.supp1 Supplementary data

10.1136/jitc-2020-001064.supp2 Supplementary data

10.1136/jitc-2020-001064.supp3 Supplementary data

**Table 2:** Best overall response (per RECIST 1.1, based on investigator assessment)

Response	N=156
Best overall response, n (%)	
Complete response	3 (1.9)
Partial response	28 (17.9)
Stable disease	68 (43.6)
Progressive disease	40 (25.6)
Not evaluable*	17 (10.9)
ORR (95% CI), %	19.9 (13.9 to 27.0)
Disease control rate, n (%)	99 (63.4)

*Includes patients with no post-baseline assessment (n=9); stable disease of insufficient duration (<6 weeks without further assessment available; n=4); new therapy started before first post-baseline assessment (n=2); non-evaluable assessments (n=1); or non-evaluable with progressive disease occurring >12 weeks after study assessment (n=1).

ORR, objective response rate; RECIST, Response Evaluation Criteria in Solid Tumors.

In analyzes of efficacy in subgroups with PD-L1 expression levels of ≥1%, ≥50%, and ≥80%, ORRs by RECIST 1.1 were 19.3% (95% CI, 11.7% to 29.1%), 22.6% (95% CI, 12.3% to 36.2%), and 26.3% (95% CI, 13.4% to 43.1%), respectively (table 3). Patients who were not evaluable for PD-L1 status had an ORR of 26.7% (95% CI, 14.6% to 41.9%). Best change in target lesions by PD-L1 status is shown in online additional file 2. In PD-L1+ and PD-L1− subgroups (≥1% and <1% cut-offs, respectively), median PFS was 4.0 months (95% CI, 2.7 to 6.0 months) and 1.5 months (95% CI, 1.35 to 5.4 months), and median OS was 14.1 months (95% CI, 11.2 to 18.2 months) and 11.3 months (95% CI, 1.6 to not estimable), respectively.

**Table 3:** ORR according to tumor PD-L1 status (cut-off indicates percentage of tumor cells expressing PD-L1)

	PD-L1 positive	PD-L1 negative	P value
≥1% cut-off			
Patients, n	88	23	–
ORR (95% CI), %	19.3 (11.7 to 29.1)	8.7 (1.1 to 28.0)	0.353
Median PFS (95% CI), months	4.0 (2.7 to 6.0)	1.5 (1.4 to 5.4)	–
Median OS (95% CI), months	14.1 (11.2 to 18.2)	11.3 (1.6 to NE)	–
≥50% cut-off			
Patients, n	53	58	–
ORR (95% CI), %	22.6 (12.3 to 36.2)	12.1 (5.0 to 23.3)	0.207
Median PFS (95% CI), months	5.4 (2.8 to 9.6)	2.4 (1.4 to 2.8)	–
Median OS (95% CI), months	14.2 (11.9 to NE)	13.6 (6.8 to 18.2)	–
≥80% cut-off			
Patients, n	38	73	–
ORR (95% CI), %	26.3 (13.4 to 43.1)	12.3 (5.8 to 22.1)	0.109
Median PFS (95% CI), months	5.4 (2.7 to 11.1)	2.7 (1.4 to 4.2)	–
Median OS (95% CI), months	14.2 (12.4 to 16.9)	14.0 (8.4 to 19.7)	–

NE, not estimable (not reached); ORR, objective response rate; OS, overall survival; PD-L1, programmed death ligand 1; PFS, progression-free survival.

In analyzes of response by immune-related criteria, four additional patients, who did not achieve a response according to RECIST 1.1, had an objective response, resulting in an immune-related ORR of 22.4%, including immune-related CRs in four patients (2.6%) and immune-related PRs in 31 patients (19.9%). In PD-L1 subgroups with expression levels of ≥1%, ≥50%, and ≥80%, immune-related ORRs were 23.9% (95% CI, 15.4% to 34.1%), 28.3% (95% CI, 16.8% to 42.3%), and 34.2% (95% CI, 19.6% to 51.4%), respectively. In the overall population, median PFS based on immune-related criteria was 6.9 months (95% CI, 5.4 to 9.7 months).

### Safety

#### AEs (irrespective of relationship to treatment)

In total, 156 patients (100%) had an AE of any grade. AEs led to permanent treatment discontinuation in 31 patients (19.9%). Twenty patients (12.8%) died following an AE that was unrelated to treatment; no deaths were considered related to treatment. IRR, identified using an expanded definition, was the most common AE and occurred in 40 patients (25.6%), including grade ≥3 AEs in five patients (3.2%). Most IRRs (34 of 40 patients) occurred after the first infusion, and eight patients (5.1%) permanently discontinued treatment because of an IRR.

#### Treatment-related AEs

Treatment-related AEs (TRAEs) of any grade occurred in 107 patients (68.6%; table 4). Of 19 patients (12.2%) who had a grade ≥3 TRAE, three (1.9%) had a grade 4 TRAE (IRR, pneumonitis, and acute respiratory distress syndrome, which each occurred in one patient (0.6%)). The only grade 3 TRAEs that occurred in >1 patient were IRR (5 (3.2%)) and fatigue (4 (2.6%)). Seventeen patients (10.9%) had a TRAE that led to permanent treatment discontinuation (online additional file 4).

10.1136/jitc-2020-001064.supp4 Supplementary data

**Table 4:** TRAEs (any grade in ≥5% of patients or grade ≥3 in any patient) and IRRs

	N=156	
	Any grade	Grade ≥3
Any TRAE, n (%)*	107 (68.6)	19 (12.2)
Fatigue	32 (20.5)	4 (2.6)
Nausea	19 (12.2)	0
Hypothyroidism	14 (9.0)	0
Diarrhea	12 (7.7)	0
Chills	11 (7.1)	0
Decreased appetite	10 (6.4)	1 (0.6)
Arthralgia	9 (5.8)	0
Dry skin	9 (5.8)	0
Pruritus	8 (5.1)	0
Fever	8 (5.1)	0
Vomiting	8 (5.1)	0
Pneumonitis	5 (3.2)	1 (0.6)
Lipase increased	4 (2.6)	1 (0.6)
Hypokalemia	2 (1.3)	1 (0.6)
Hyponatremia	2 (1.3)	1 (0.6)
Acute respiratory distress syndrome	1 (0.6)	1 (0.6)
Endocrine disorder	1 (0.6)	1 (0.6)
Hypertension	1 (0.6)	1 (0.6)
Hypoxia	1 (0.6)	1 (0.6)
Musculoskeletal chest pain	1 (0.6)	1 (0.6)
Nephrotic syndrome	1 (0.6)	1 (0.6)
Pneumothorax	1 (0.6)	1 (0.6)
IRR, n (%)†	40 (25.6)	5 (3.2)

*Incidence of treatment-related IRR based on the single MedDRA preferred term is not listed.

†Composite term, which includes AEs categorized as IRR, drug hypersensitivity, or hypersensitivity reaction that occurred on the day of or day after infusion, in addition to signs and symptoms of IRR that occurred on the same day of infusion and resolved within 2 days (including AEs classified by investigators as related or unrelated to treatment).

AE, adverse event; IRR, infusion-related reaction; MedDRA, Medical Dictionary for Regulatory Activities; TRAE, treatment-related adverse event.

#### Serious TRAEs

Serious TRAEs occurred in 15 patients (9.6%), and the most common (≥2 patients) were IRR (4 (2.6%)) and pneumonitis (2 (1.3%)).

#### Immune-related adverse events

Thirty-one patients (19.9%) had an irAE, of which one patient (0.6%) had a grade ≥3 irAE (pneumonitis; table 5). The most common category was endocrine irAEs, which included 15 patients (9.6%) with either hypothyroidism or hyperthyroidism, and one patient (0.6%) with adrenal insufficiency. In addition, cutaneous irAEs, including immune-mediated rash or pruritus, occurred in 12 patients (7.7%), pneumonitis occurred in five patients (3.2%), and immune-mediated diarrhea or colitis occurred in three patients (1.9%).

**Table 5:** Immune-related adverse events (any grade in any patient; n=156)

	Patients, n (%)	
	Any grade	Grade ≥3
Any immune-related adverse event	31 (19.9)	1 (0.6)
Immune-mediated thyroid disorder	15 (9.6)	0
Hypothyroidism	14 (9.0)	0
Hyperthyroidism	1 (0.6)	0
Immune-mediated rash or pruritus	12 (7.7)	0
Pruritus	6 (3.8)	0
Rash	6 (3.8)	0
Rash maculopapular	2 (1.3)	0
Pruritus generalized	1 (0.6)	0
Rash erythematous	1 (0.6)	0
Rash macular	1 (0.6)	0
Immune-mediated pneumonitis	5 (3.2)	1 (0.6)
Immune-mediated colitis	3 (1.9)	0
Diarrhea	2 (1.3)	0
Colitis	1 (0.6)	0
Immune-mediated adrenal insufficiency	1 (0.6)	0
Autoimmune disorder	1 (0.6)	0

## Discussion

In this study, first-line avelumab monotherapy showed clinical activity and an acceptable safety profile in patients with treatment-naive advanced NSCLC. The ORR was 19.9% and the median DOR was 12.0 months. In comparison, ORRs in studies of avelumab in patients with platinum-treated advanced NSCLC, who were unselected for PD-L1 status, were 14% in a separate phase I cohort of the JAVELIN Solid Tumor trial^22^ and 15% in the phase III JAVELIN Lung 200 trial.^23^ In the current study, patients with ≥50% and ≥80% PD-L1-positive tumors had ORRs (by RECIST 1.1) of 22.6% and 26.3%, respectively. However, a high proportion of patients (28.8%) were not evaluable for tumor PD-L1 expression, and patient numbers were low, particularly in the high PD-L1-positive subgroup (n=38), which hampers interpretation of the biomarker data. In the overall population, the median PFS was 4.0 months and the median OS was 14.1 months. Response rates and PFS assessed using immune-related criteria were slightly increased compared with analyzes based on RECIST 1.1. Although the study included small subgroups with unknown *EGFR* and *ALK* mutation status, ORRs in these patients were similar to the ORR in the overall population. Safety outcomes were comparable to previous studies of avelumab and other anti-PD-1/PD-L1 agents in NSCLC.^4 12 13 22 24–26^

Data from this study can be considered in the context of similar early-phase studies of other anti-PD-1/PD-L1 antibodies administered as first-line monotherapy for PD-L1-positive NSCLC, although cross-trial comparisons should be interpreted with caution as eligibility criteria and patient populations may differ, and companion assays to detect PD-L1 expression were developed independently for each agent. In a cohort of a phase I study (KEYNOTE-001) in which patients with treatment-naive NSCLC received pembrolizumab, the ORR was 27%, median PFS was 6.2 months, and median OS was 22.1 months; efficacy was increased in patients whose tumors had high PD-L1 expression (≥50% of tumor cells PD-L1+ using the 22C3 assay, which is comparable to ≥80% of tumor cells PD-L1+ using the more sensitive 73-10 assay).^24 27^ In patients with NSCLC treated with first-line nivolumab in the phase I CheckMate 012 trial, the ORR was 23%, median PFS was 3.6 months, and median OS was 19.4 months.^25^ In addition, in patients with PD-L1-high NSCLC (≥25% PD-L1 expression on tumor cells; SP263 assay) treated with first-line durvalumab in the phase I/II 1108 study, the ORR was 27%, median PFS was 5.4 months, and median OS was 21.9 months.^26^ Subsequent phase III trials of anti-PD-1/PD-L1 antibodies versus platinum-based chemotherapy in the first-line NSCLC setting have produced conflicting findings. In KEYNOTE-024 and KEYNOTE-042, pembrolizumab showed superior OS versus platinum-based chemotherapy in patients with advanced NSCLC with ≥50% and ≥1% PD-L1 expression on tumor cells, respectively,^2 3^ which provided the basis for the approval of pembrolizumab in this setting. Similarly, in the recently reported IMpower110 trial, first-line atezolizumab showed superior OS versus platinum-based chemotherapy in patients with PD-L1-high NSCLC (PD-L1 expression on ≥50% of tumor cells and/or ≥10% of tumor-infiltrating immune cells; SP142 assay).^28^ However, in CheckMate 026, nivolumab did not show superior OS versus platinum-based chemotherapy in patients with PD-L1-positive tumors (≥5% PD-L1 expression on tumor cells; 28-8 assay).^4^ Similarly, in the MYSTIC trial, durvalumab alone or in combination with tremelimumab (anticytotoxic T-lymphocyte-associated protein 4) was not superior to platinum-based doublet chemotherapy in patients with PD-L1-high NSCLC (≥25% PD-L1 expression on tumor cells; SP263 assay).^29^ More recently, several phase III trials have reported superior efficacy for anti-PD-1 or anti-PD-L1 antibodies combined with chemotherapy versus chemotherapy alone in NSCLC irrespective of PD-L1 expression.^5–7^ However, combination regimens may be associated with increased toxicity burden; thus checkpoint inhibitor monotherapy with pembrolizumab remains a standard first-line treatment for PD-L1-high NSCLC.^30 31^ The ongoing phase III JAVELIN Lung 100 study (NCT02576574), which was initiated in 2015, is assessing first-line avelumab monotherapy compared with platinum-based doublet chemotherapy in patients with PD-L1-positive NSCLC. The primary analysis population in the JAVELIN Lung 100 study consists of patients with high PD-L1-expressing tumors (≥80% of tumor cells; 73-10 assay); hence, this study will provide an assessment of avelumab in a patient population similar to those of earlier trials of anti-PD-1/PD-L1 monotherapy.

In summary, the results from this phase Ib study showed that avelumab monotherapy has clinical activity and acceptable safety as a first-line treatment for patients with advanced NSCLC, providing the rationale for further studies. Findings from the phase III JAVELIN Lung 100 study will help clarify the potential role of avelumab monotherapy in the NSCLC treatment landscape.
