# Supplementary material for: Efficacy and safety of first-line avelumab in patients with advanced non-small cell lung cancer: results from a phase Ib cohort of the JAVELIN Solid Tumor study
Source: J Immunother Cancer. 2020 Sep 8;8(2):e001064. doi: 10.1136/jitc-2020-001064 (PMC7481079; doi:10.1136/jitc-2020-001064)
Supplement: Supplementary data [file jitc-2020-001064supp001.pdf]

Additional file 1. Percentage change from baseline in target lesions over time in evaluable patients (n = 142).

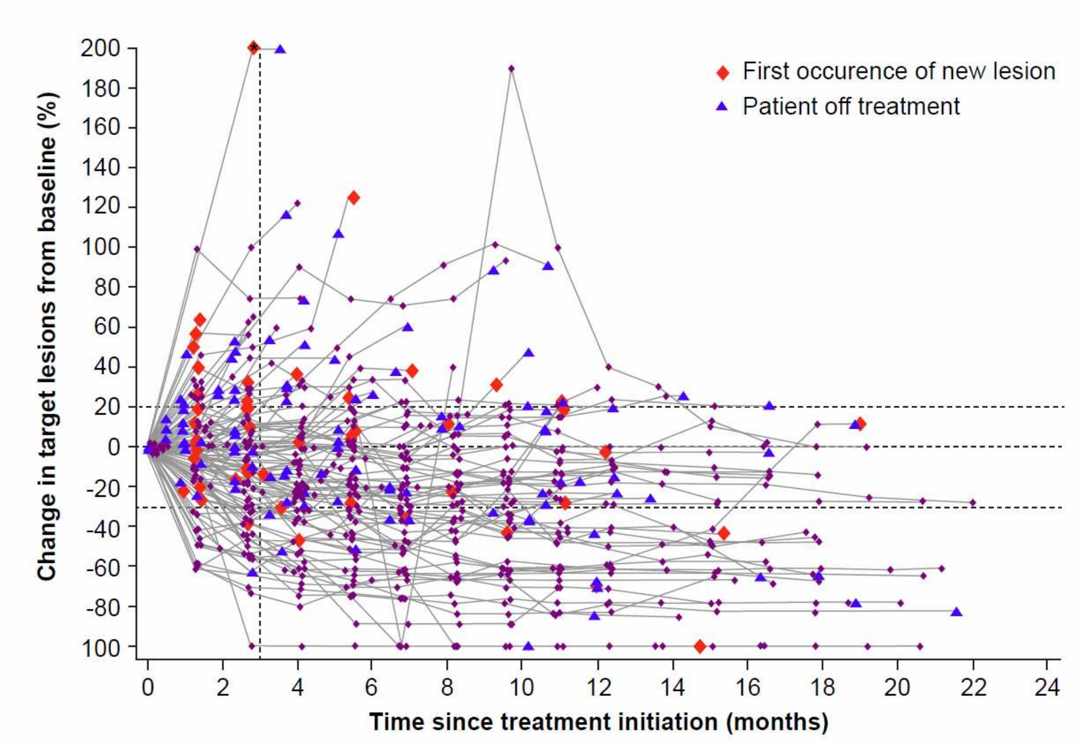

*Data point imputed with a cap of 200%.

Verschraegen CF, *et al. J Immunother Cancer* 2020; 8:e001064. doi: 10.1136/jitc-2020-001064
