# Supplementary material for: Efficacy and safety of first-line avelumab in patients with advanced non-small cell lung cancer: results from a phase Ib cohort of the JAVELIN Solid Tumor study
Source: J Immunother Cancer. 2020 Sep 8;8(2):e001064. doi: 10.1136/jitc-2020-001064 (PMC7481079; doi:10.1136/jitc-2020-001064)
Supplement: Supplementary data [file jitc-2020-001064supp002.pdf]

Additional file 2. Best change from baseline in target lesions in evaluable patients (patients with a baseline and at least 1 post-baseline lesion assessment; n = 142) by (**A**) PD-L1 expression, (**B**) tumor histology, and (**C**) smoking status.

A.

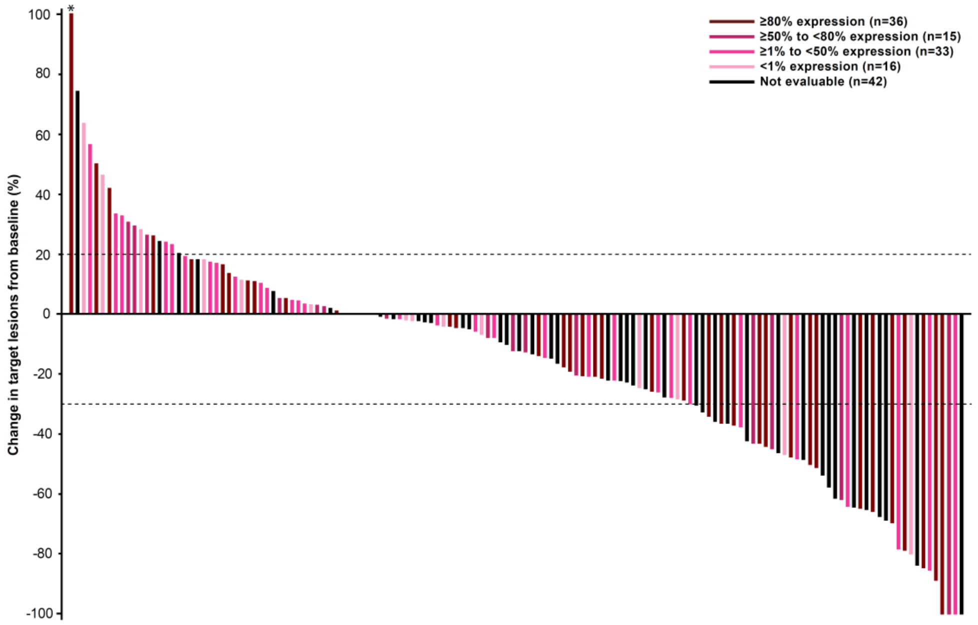

B.

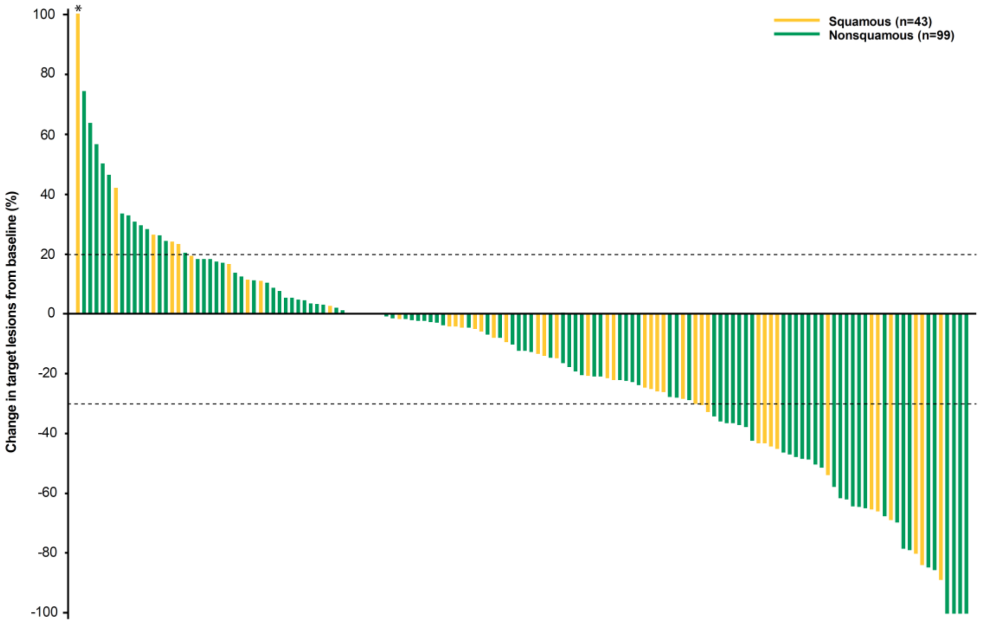

C.

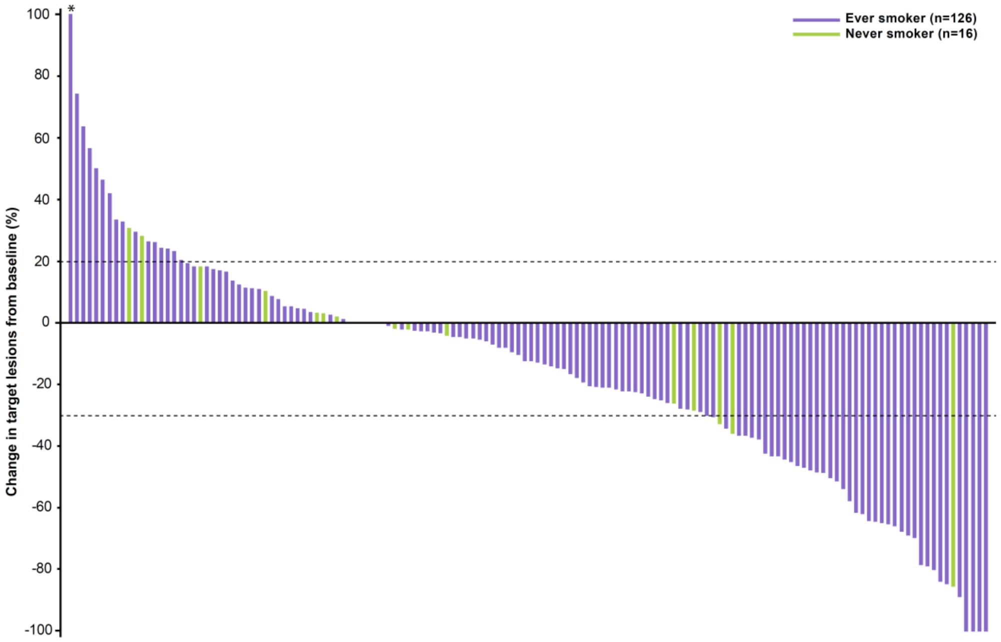

* Patient with a 415% increase in tumor diameter imputed with a cap of 100%.
