# Supplementary material for: Efficacy and safety of first-line avelumab in patients with advanced non-small cell lung cancer: results from a phase Ib cohort of the JAVELIN Solid Tumor study
Source: J Immunother Cancer. 2020 Sep 8;8(2):e001064. doi: 10.1136/jitc-2020-001064 (PMC7481079; doi:10.1136/jitc-2020-001064)
Supplement: Supplementary data [file jitc-2020-001064supp003.pdf]

**Additional file 3. A.** Overall survival (OS). **B.** Progression-free survival (PFS; per RECIST 1.1). N = 156.

**A.**

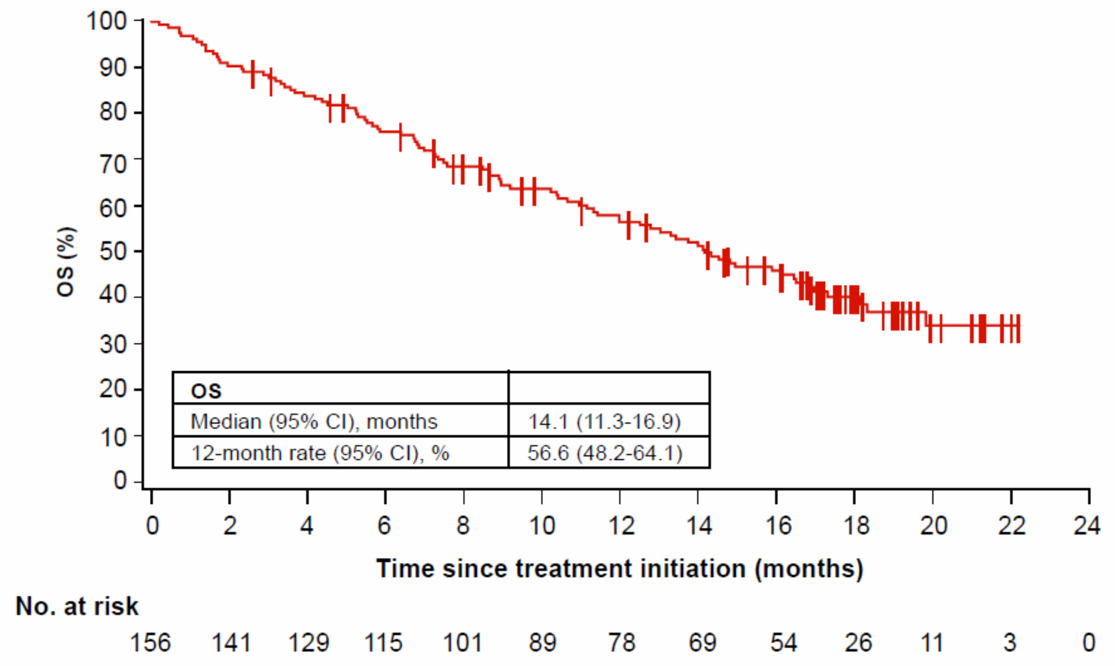


**B.**

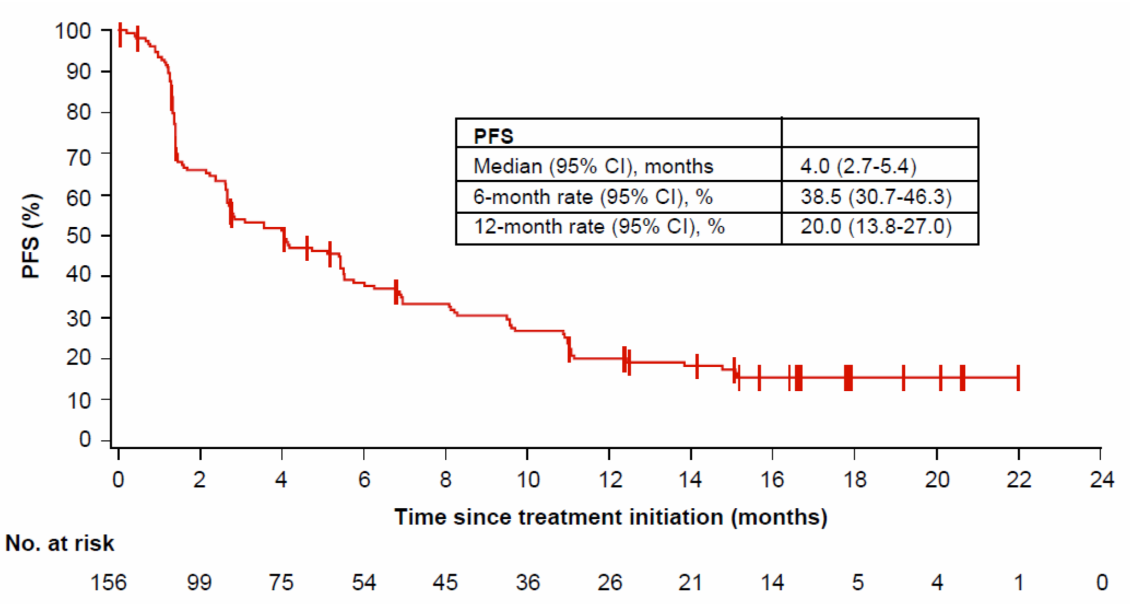

OS, overall survival; PFS, progression-free survival; RECIST, Response Evaluation Criteria in Solid Tumors.



Verschraegen CF, *et al. J Immunother Cancer* 2020; 8:e001064. doi: 10.1136/jitc-2020-001064
