# Supplementary material for: Efficacy and safety of first-line avelumab in patients with advanced non-small cell lung cancer: results from a phase Ib cohort of the JAVELIN Solid Tumor study
Source: J Immunother Cancer. 2020 Sep 8;8(2):e001064. doi: 10.1136/jitc-2020-001064 (PMC7481079; doi:10.1136/jitc-2020-001064)
Supplement: Supplementary data [file jitc-2020-001064supp004.pdf]

Additional file 4. Treatment-related adverse events (TRAEs) leading to permanent treatment discontinuation

**Adverse event**	**N=156**
TRAE leading to treatment discontinuation, n (%)	17 (10.9)
Infusion-related reaction*	8 (5.1)
Pneumonitis	2 (1.3)
Acute kidney injury	1 (0.6)
Autoimmune disorder	1 (0.6)
Blood creatinine increased	1 (0.6)
Diarrhea	1 (0.6)
Dry mouth	1 (0.6)
Dysgeusia	1 (0.6)
Fatigue	1 (0.6)
Hyperkalemia	1 (0.6)
Nausea	1 (0.6)
Nephrotic syndrome	1 (0.6)

*IRRs were identified using an expanded definition that included both a prespecified list of Medical Dictionary for Regulatory Activities preferred terms (infusion-related reaction, drug hypersensitivity, or hypersensitivity reaction) that occurred post infusion within 48 hours, and additional signs or symptoms that occurred on the day of infusion and resolved within 2 days.
Some patients had >1 TRAE that led to discontinuation.

Verschraegen CF, *et al. J Immunother Cancer* 2020; 8:e001064. doi: 10.1136/jitc-2020-001064
